# The ethical imperative to reduce HIV stigma through community‐engaged, status‐neutral interventions designed with and for transgender women of colour in the United States

**DOI:** 10.1002/jia2.25907

**Published:** 2022-07-12

**Authors:** Kristi E. Gamarel, Greg Rebchook, Breonna M. McCree, Laura Jadwin‐Cakmak, Maureen Connolly, Lilianna A. Reyes, Jae M. Sevelius

**Affiliations:** ^1^ Department of Health Behavior and Health Education University of Michigan School of Public Health Ann Arbor Michigan USA; ^2^ Division of Prevention Sciences Department of Medicine University of California San Francisco San Francisco California USA; ^3^ Department of Pediatrics Henry Ford Health System Detroit Michigan USA; ^4^ Trans Sistas of Color Project Detroit Michigan USA

**Keywords:** transgender, HIV stigma, intervention, women, HIV prevention, HIV treatment

## Abstract

**Introduction:**

In the era of biomedical HIV prevention and treatment technologies, such as treatment as prevention (TasP) and pre‐exposure prophylaxis (PrEP), there is momentum to develop and rigorously evaluate interventions focused on PrEP among those at risk for HIV acquisition and antiretroviral therapy (ART) adherence among people living with HIV. While HIV status‐specific interventions focused on PrEP or ART provide valuable information, status‐segregated interventions can create, perpetuate, and even increase HIV stigma among transgender women of colour and other marginalized communities in the United States (US).

**Discussion:**

Due largely to community advocacy, discourses that support status‐neutral approaches have emerged in the scientific literature. Although US‐based funding mechanisms have typically designated awards focused on a specific HIV status, intervention developers and implementing agencies find creative ways to design and implement status‐neutral programmes despite such restrictions. We present our experience with intervention research in New York, Detroit, New Orleans, Puerto Rico and the San Francisco Bay Area, all Ending the HIV Epidemic (EHE) priority jurisdictions. Kickin it with the Gurlz’ was developed to be status‐neutral through two grants due to community demands for a unifying approach. The Transgender Women Engagement and Entry to (TWEET) Care Project was designed to improve HIV care engagement for transgender women living with HIV, but developers realized the importance of including participants of any HIV status. Healthy Divas was designed for transgender women living with HIV but subsequent implementing agencies prioritized adapting it to be status‐neutral. These examples support the urgency of designing, implementing and evaluating status‐neutral interventions.

**Conclusions:**

Community‐based organizations strive for inclusivity in their programming and are rightly often reluctant to segregate services based on the HIV status of their clients. As researchers, we have an ethical imperative to work to reduce HIV stigma and respond to the needs of those most impacted by HIV, including transgender women of colour. As such, we call upon funders to develop mechanisms that support the development and testing of HIV status‐neutral interventions to reduce HIV stigma and support community building, thereby increasing the possibility of fully realizing the benefits of biomedical HIV prevention and treatment technologies for all.

## 1 INTRODUCTION

There have been incredible strides to prevent human immunodeficiency virus (HIV) transmission and ensure that people living with HIV live longer and healthier lives [1, 2]. In the era of biomedical technologies, such as treatment as prevention (TasP) and pre‐exposure prophylaxis (PrEP), there has been global momentum to develop and rigorously evaluate interventions focused on PrEP among those at risk for HIV acquisition and antiretroviral therapy (ART) adherence among people living with HIV. While interventions focused on PrEP or ART uptake and adherence have and will continue to benefit communities, these HIV status‐specific or what we have termed “status‐segregated” interventions can perpetuate HIV stigma and other forms of oppression among those in most need of HIV programmes [3], especially among transgender women of colour, by inadvertently disclosing HIV status to community members [4], allocating scarce resources to members of a community with a specific HIV status [5] and can increase HIV stigma and reduce social support by imposing artificial divisions in the community [6].

In the United States (US), transgender women of colour have expressed the urgency of grounding HIV prevention and care programming in their lived experiences, noting that oppression rooted in ethno‐racism and cisgenderism and resultant social determinants of health (e.g. unmet gender affirmation needs, biased policing and hyper‐surveillance that results in overrepresentation in the legal‐criminal system, economic vulnerability and immigration experiences) take precedence over HIV [7, 8, 9, 10, 11, 12]. For example, one young transgender woman of colour described the importance of addressing social determinants of HIV: “We need jobs, places to stay, doctors. HIV is just one of the many problems we deal with” [13]. Further, segregating people into HIV prevention and HIV treatment research disrupts the organic and close kinship structures among transgender women of colour that are needed to buffer experiences of oppression based on ethno‐racism and cisgenderism [5]. Status‐segregated intervention research can feel forced, divisive and culturally unresponsive to the needs and experiences of transgender women of colour [5, 11]. That is, dividing community by HIV status can perpetuate HIV stigma among close‐knit marginalized communities when resources are already scarce [5].

Evidence also suggests that HIV status‐neutral interventions are preferred in community‐based settings [14, 15], as programme participants can address shared experiences of intersectional oppression, structural determinants of HIV and mental health challenges driving HIV risk and suboptimal HIV prevention and treatment uptake, and decrease HIV stigma. These interventions are urgently needed to optimize HIV prevention and care outcomes with transgender women of colour [16]. To meet this need, community‐based organizations often adapt evidence‐based interventions that were designed to be status‐segregated to be status‐neutral. While these status‐neutral approaches are more responsive to their clients as well as their implementation context, these adaptations are being implemented without rigorous evaluation [17]. Building on Myers and colleagues’ status‐neutral framework, we present evidence from ongoing intervention research in New York, Detroit, New Orleans, Puerto Rico and the San Francisco Bay Area, all Ending the HIV Epidemic (EHE) priority jurisdictions, supporting the utility of designing and implementing status‐neutral interventions with transgender women of colour (Table 1).

**Table 1:** HIV intervention approaches for transgender women of colour in the United States

Intervention	Eligibility criteria	Theoretical framework	Intervention components	Location
TWEET Original	At least 18 years old	Social cognitive theory	Transgender leaders teach back groups led by peer leaders	Detroit, Michigan, New Orleans, Louisiana, Ponce, Puerto Rico, Queens, New York
	Assigned male at birth and currently identifies as female, trans female, trans sexual and transgender	Social learning theory	Community outreach and recruitment	
	Fluent in English or Spanish	Trans‐theoretical model	Supportive retention services (e.g. assistance with name change, gender markers, gender affirming care referrals; patient navigation and benefits counselling; referrals to comprehensive legal services)	
	Identifies as one or more of the following racial/ethnic categories: Hispanic/Latino/Puerto Rican/Cuban, Black or African American, American Indian or Alaska Native, Asian Indian, Chinese, Filipino, Japanese, Korean, Vietnamese, Other Asian, Native Hawaiian, Guamanian or Chamorro, Samoan, or Other Pacific Islander Living with HIV to be included in the cross‐site evaluation. Intervention activities were open to any HIV status			
TWEET Implementation	At least 18 years old	Social cognitive theory	Transgender leaders teach back groups led by peer leaders	
	Assigned male at birth and currently identifies as female or trans female	Trans‐theoretical model	Community outreach and recruitment	
	Fluent in English or Spanish		Supportive retention services (e.g. assistance with name change, gender markers, gender affirming care referrals; patient navigation and benefits counselling; referrals to comprehensive legal services)	
	Living with HIV to be included in the cross‐site evaluation. Intervention activities were open to any HIV status			
Healthy Divas RCT	At least 18 years old	Gender affirmation model	Six peer‐led individual sessions, held weekly, and one group workshop facilitated by a healthcare provider with expertise in HIV care and transgender health	San Francisco and Los Angeles, California
	Assigned male at birth and currently identifies as female or trans female, or another transfeminine identity	Healthcare empowerment model		
	Fluent in English or Spanish			
	Living with HIV, as confirmed be antibody testing			
	Reports suboptimal engagement in HIV care, as indicated by one or more of the following: (1) not on ART, (2) if on ART, reported less than perfect adherence on a validated adherence rating scale, 38 or (3) reported no HIV primary care appointments in the prior 6 months			
Healthy Divas Implementation Study	At least 18 years old	Gender affirmation model	Expanded on Healthy Divas intervention content (described above) to be status neutral and include information relevant to trans women of negative or unknown HIV status	Oakland, California
	Assigned male at birth and currently identifies as female, trans female or another transfeminine identity	Healthcare empowerment model		
	Fluent in English or Spanish			
Kickin’ it with Gurlz	At least 18 years old	Gender affirmation model	Integration of three evidence‐based interventions: (1) gender affirmation and safety needs screening; (2) at least two peer navigation sessions; and (3) eight peer‐delivered culturally adapted group sessions based in cognitive behavioural therapy strategies from the Seeking Safety programme with an explicit focus on intersectional oppression and resistance	Detroit, Michigan
	Assigned male at birth and identifies as female, transgender woman or another feminine gender identity	Critical consciousness		
	Self‐identifies as a person of colour (any racial/ethnic identity except non‐Hispanic white)			
	History of trauma (i.e. endorses at least two items on the adapted Trauma History Screener, which includes IPV and experiencing or witnessing other forms of violence)			
	Living or willing to travel to Detroit			
	English speaking			
TRIUMPH	18 years or older	Gender affirmation model	Peer health education, peer‐led community mobilization and clinical integration of PrEP with hormone therapy to promote PrEP knowledge and acceptability	Oakland and Sacramento, California
	HIV negative (confirmed by rapid test)			
	Report a gender identity different from the sex assigned at birth			
	Currently sexually active or intending to become sexually active, express a desire to use Prep			
	Fluent in English or Spanish			
Triunfo (TRIUMPH) Implementation Study	At least 18 years old	Gender affirmation model	Expanded on TRIUMPH intervention (described above) to include peer health education and navigation to services relevant to trans women living with HIV	Oakland, California
	Assigned male at birth and currently identifies as female, trans female or another transfeminine identity			
	Fluent in English or Spanish			

Abbreviations: ART, antiretroviral therapy; IPV, interpersonal violence; PrEP, pre‐exposure prophylaxis.

## 2 DISCUSSION

Even though US federal funding mechanisms often designate awards to focus on people of a specific HIV status, intervention developers and implementing agencies can and do find creative ways to design and implement status‐neutral programmes despite such restrictions. For example, the Health Resources and Services Administration (HRSA) funded the Enhancing Engagement and Retention in Quality HIV Care for Transgender Women of Colour Initiative in 2012. This Special Projects of National Significance (SPNS) initiative supported nine demonstration projects to develop and evaluate innovative interventions to reduce HIV health inequities among transgender women of colour living with HIV [18]. As part of this initiative, the Community Healthcare Network (CHN) in Queens, New York developed and tested the Transgender Women Engagement and Entry To (TWEET) Care Project, a peer‐led, group‐based educational intervention [19]. During the TWEET development process, CHN realized that to deal with HIV stigma in their community, they needed to include transgender women of any HIV status, such that participating in TWEET did not “out” the participants as living with HIV. As stated by the TWEET developer, it “created a welcoming supportive environment to address HIV stigma. The transgender women in the programme who were living with HIV were able to share their experiences on how they coped with their HIV status, which in turn served as an educational tool for those who were not living with HIV.” (Personal Communication with Luis Freddy Molano MD, August 2020). To meet funding requirements, only transgender women living with HIV were included in the initiative's cross‐site evaluation, and SPNS resources were not used to incentivize participation for participants not living with HIV.

Three organizations replicating TWEET in HRSA's E2i Initiative (Using Evidence‐Informed Interventions to Improve Health Outcomes among People Living with HIV) [20] also adopted CHN's status‐neutral approach to TWEET by including transgender women of any HIV status in response to HIV stigma in Ponce, Puerto Rico, New Orleans, Louisiana, Los Angeles, California and Detroit, Michigan. TWEET in these three communities was able to create social cohesion between transgender women regardless of HIV status, and the status‐neutral approach facilitated participant recruitment by not requiring participants to self‐identify as living with HIV to be included in the TWEET groups. Importantly, many individuals *chose* to share their HIV status over the course of the project, which fostered self‐efficacy in communicating about HIV, allowed group members to form deeper connections with each other and provided an opportunity to collectively challenge prevailing HIV stigma. In Detroit, TWEET has continued to be implemented as a peer‐delivered status‐neutral intervention even after the end of the funding to meet the community demand for such a unifying and supportive group led by transgender women of colour.

Beyond TWEET, status‐neutral interventions that simultaneously address trauma are urgently needed in Detroit. As Michigan's HIV epicentre [21], patterns of economic disadvantage, racial segregation and anti‐transgender stigma have resulted in limited engagement in both HIV prevention and care among transgender women of colour [22]. We applied to a request for applications to the US National Institutes of Health that was focused on addressing violence along the HIV care continua among transgender women of colour. The few existing services in Detroit are primarily funded by HIV dollars to support people living with HIV. Although these behavioural programmes focused on HIV care are critical, with few exceptions (e.g. TWEET) they have historically excluded transgender women of colour not living with HIV who need these services and inadvertently disclosed programme participants’ HIV status [5]. At the time of writing the grant proposal in Detroit, we knew we would need to find additional funds to include transgender women not living with HIV given the documented need for status‐neutral programmes. Fortunately, we were awarded the grant to develop and pilot a multicomponent intervention designed to help transgender women of colour living with HIV heal from violence and trauma. After many conversations, we applied for and received an additional grant to develop and pilot the intervention with transgender women of colour not living with HIV. Although funded at the start of the COVID‐19 pandemic, the status‐neutral approach named “Kickin it with the Gurlz” by our team of Latina and Black transgender women in Detroit has been a success. Across interviews (*n* = 11) and focus groups (*n* = 12) to adapt the intervention content, participants expressed a desire for status‐neutral groups citing the importance of “breaking down stigma in community.” While only possible through two separate funding mechanisms, our team is in a position to examine the feasibility and acceptability of a status‐neutral intervention with transgender women of colour designed to address both HIV prevention and care continua outcomes at the outset.

In San Francisco, the UCSF Center of Excellence for Transgender Health (UCSF CoE) has spent the better part of a decade developing and testing a peer‐led, gender‐affirming intervention for transgender women living with HIV, called Healthy Divas. Significant funding and resources have been invested in pilot testing and conducting a randomized controlled trial of the intervention (R01MH106373). Based on the urgent need for such a programme, Healthy Divas was selected for national dissemination by HRSA's E2i Initiative [20]. Further, as part of our UCSF Prevention Research Center's core research activities, we are conducting an implementation study of Healthy Divas with Cal‐PEP, a community‐based organization serving African American transgender women communities in Oakland, California. As part of this implementation study, Cal‐PEP expressed a strong preference to adapt Healthy Divas to be status‐neutral, similar to the approach used in the TWEET original research and subsequent replication. Additionally, Cal‐PEP was facing recruitment barriers due to the fact that the programme is known to serve people living with HIV and may inadvertently disclose a client's HIV status to the close‐knit Cal‐PEP community. These concerns were so urgent and valid that, in collaboration with Cal‐PEP, the UCSF CoE rapidly adapted the intervention's content to include transgender women who are not living with HIV, incorporated information about HIV testing and PrEP in the curriculum and re‐trained the peer facilitators to implement the status‐neutral version of Healthy Divas. As a peer counselling and client‐centred intervention, Healthy Divas was designed to be flexible and adaptive to the concerns of the client and was, therefore, amenable to adaptation. However, without adequate testing, we are unsure of the impact this adaptation might have on the intervention's efficacy.

The UCSF CoE has also been conducting PrEP research with Latina transgender women at La Clínica de la Raza, a community‐based clinic in a predominantly Latinx neighbourhood of Oakland, California. Triunfo was designed as a peer‐led, PrEP education and community mobilization project to encourage PrEP uptake and adherence among Latina transgender women at risk for HIV acquisition. As a result of the focus on those not living with HIV, anyone in the community who was not able to participate in Triunfo was outed as living with HIV, which resulted in inadvertent reinforcement of HIV stigma and a sense of the programme being unnecessarily exclusionary and divisive. Our university‐academic partnership recently received funding to expand the programme at La Clinica to include Latina transgender women living with HIV and study the implementation of a status‐neutral version of the intervention designed to improve both HIV prevention and HIV treatment.

## 3 CONCLUSIONS

With evidence and advocacy to support HIV status‐neutral approaches as a means to eradicate HIV stigma globally [23, 24, 25], it is essential that federal funding, such as the recent request for applications by the US Centers for Disease Control (CDC‐RFA‐PS22‐2209) and US National Institutes of Health (PAR‐21‐344) focused on low‐ and middle‐income countries, provides mechanisms for developing and testing these approaches. Such funding avenues have the potential to result in better science and implementation outcomes in a context in which community‐based organizations are currently implementing adapted versions of evidence‐based interventions that have not been rigorously tested. Additionally, since evidence‐based interventions only have an impact on the HIV epidemic when they are implemented, it is important that these interventions are consistent with the organizational culture, mission and value systems of community‐based agencies. Status‐segregated interventions can run counter to the needs of many such agencies to create connections across multiple intersections, including HIV status.

Social support, community connection and social capital are important mechanisms that reduce the deleterious impact of HIV stigma on HIV care continua outcomes, including engagement in care and viral suppression [26, 27, 28]. There have been a handful of promising status‐neutral interventions designed with and for transgender women to increase social support, community connection and increase access to resources. For example, LifeSkills, Couples HIV Intervention Program (CHIP) and Sheroes were designed as status‐neutral approaches, all of which were acceptable and feasible; however, the outcomes of these studies relied on self‐report and precluded biomedical confirmation (e.g. PrEP adherence and viral load) [29, 30, 31]. Additionally, there are several HIV status‐neutral interventions developed with community‐based organizations that were designed for and by transgender women of colour across the US, including programmes designed by La Clinica del Pueblo in Washington, DC and those developed for Latina trans women at TransLatin@ Coalition. The gold standard for HIV research within the scientific community now requires biomedical confirmation of self‐reported behaviours. As highlighted by Myers and colleagues, future efforts are required to consider eligibility criteria and outcome measurement using a status‐neutral approach [3]. This advancement will require working closely with community members to secure trust in collecting biomedical samples due to historical and ongoing systemic ethno‐racism and cisgenderism and ensuring that we do not place too much burden on study participants without adequate compensation. Additionally, we must develop procedures that ensure HIV status is not inadvertently disclosed in the data collection process.

HIV status‐neutral interventions have tremendous potential to reduce HIV stigma through building solidarity and information exchange among trusted peers, as well as addressing pressing needs that undermine successful engagement across both the HIV prevention and care continua [3]. However, HIV status‐neutral approaches warrant careful planning with community partners and consideration of the local context. For example, there may be additional types of support needed for HIV disease management and living with a chronic illness compared to HIV prevention programmes. While there may continue to be a need for status‐segregated programming, HIV stigma is pervasive and status‐neutral approaches can serve as platform for deconstructing HIV stigma and avoid the victim‐centric approach that has historically predominated existing individual‐level interventions for people living with HIV [32]. There may also be concerns that status‐neutral approaches may divert funds from people living with HIV. Our intention is not to advocate for reduced funding or compromise quality of care for people living with HIV but rather to increase funding for all communities who experience intersectional oppression to reduce HIV inequities.

Community‐based organizations strive for inclusivity in their programming and are reluctant to segregate their services based on HIV status. We have an ethical imperative to respond to the needs of those most impacted by HIV, specifically transgender women of colour. Our HIV intervention efforts must be designed to decrease or eliminate HIV stigma among marginalized communities who experience multiple and interlocking systems of oppression. Funding priorities focused exclusively on status‐segregated intervention research create an ethical dilemma for HIV researchers wishing to collaborate effectively with communities most impacted by HIV. As such, we call upon funders to develop funding mechanisms that support the development and testing of HIV status‐neutral intervention research to reduce HIV stigma, support community building and fully realize the benefits of biomedical HIV prevention and treatment technologies for all.

## COMPETING INTERESTS

The authors declare no competing interests.

## AUTHORS’ CONTRIBUTIONS

KEG, GR and JMS wrote the first draft of the manuscript. BMM, LJC, LAR and MC contributed to the refinement and presentation of the interventions and programmes presented. All authors contributed to the writing of the manuscript, and all approved the final draft.

## References

[1] Eisinger RW , Dieffenbach CW , Fauci AS . HIV viral load and transmissibility of HIV infection: undetectable equals untransmittable. JAMA. 2019;321(5):451–2. 30629090 10.1001/jama.2018.21167

[2] Deutsch MB , Glidden DV , Sevelius J , Keatley J , McMahan V , Guanira J , et al. HIV pre‐exposure prophylaxis in transgender women: a subgroup analysis of the iPrEx trial. Lancet HIV. 2015; 2(12):e512–9. 26614965 10.1016/S2352-3018(15)00206-4 PMC5111857

[3] Myers JE , Braunstein SL , Xia Q , Scanlin K , Edelstein Z , Harriman G , et al. Redefining prevention and care: a status‐neutral approach to HIV in open forum infectious diseases. Oxford University Press US; 2018. 10.1093/ofid/ofy097 PMC6016418 29977957

[4] Dubé K , Kanazawa J , Campbell C , Boone CA , Maragh‐Bass AC , Campbell DM , et al. Considerations for increasing racial, ethnic, gender, and sexual diversity in HIV cure‐related research with analytical treatment interruptions: a qualitative inquiry. AIDS Res Hum Retroviruses. 2022; 38(1):50–63. 33947268 10.1089/aid.2021.0023 PMC8785755

[5] Lacombe‐Duncan A , Jadwin‐Cakmak L , Trammell R , Burks C , Rivera B , Reyes L , et al. “… Everybody Else Is More Privileged. Then It's Us…”: a qualitative study exploring community responses to social determinants of health inequities and intersectional exclusion among trans women of color in Detroit, Michigan. Sex Res Soc Policy. 2021:1–21. 10.1007/s13178-021-00642-2

[6] Logie CH , Lacombe‐Duncan A , Brien N , Jones N , Lee‐Foon N , Levermore K , et al. Barriers and facilitators to HIV testing among young men who have sex with men and transgender women in Kingston, Jamaica: a qualitative study. J Int AIDS Soc. 2017; 20(1):21385. 28406274 10.7448/IAS.20.1.21385 PMC5515029

[7] Yarbrough D . The carceral production of transgender poverty: how racialized gender policing deprives transgender women of housing and safety. Punish Soc. 2021. 10.1177/14624745211017818

[8] Palazzolo SL , Yamanis TJ , Jesus MD , Maguire‐Marshall M , Barker SL . Documentation status as a contextual determinant of HIV risk among young transgender Latinas. LGBT Health. 2016; 3(2):132–8. 26669583 10.1089/lgbt.2015.0133 PMC4841909

[9] Dowshen N , Lee S , Franklin J , Castillo M , Barg F . Access to medical and mental health services across the HIV care continuum among young transgender women: a qualitative study. Transgend Health. 2017; 2(1):81–90. 28861551 10.1089/trgh.2016.0046 PMC5548410

[10] Akinola M , Wirtz AL , Chaudhry A , Cooney E , Reisner SL . Perceived acceptability and feasibility of HIV self‐testing and app‐based data collection for HIV prevention research with transgender women in the United States. AIDS Care. 2021; 33(8):1079–87. 33487032 10.1080/09540121.2021.1874269 PMC8298585

[11] Poteat T , Mayo‐Wilson LJ , Pereira N , Wright BN , Smout SA , Sawyer AN , et al. US transgender women's preferences for microeconomic interventions to address structural determinants of HIV vulnerability: a qualitative assessment. BMC Public Health. 2021; 21(1):1–15. 34261464 10.1186/s12889-021-11471-8 PMC8281671

[12] Reisner SL , Reisner SL , Chaudhry A , Cooney E , Garrison‐Desany H , Juarez‐Chavez E , et al. ‘It all dials back to safety’: a qualitative study of social and economic vulnerabilities among transgender women participating in HIV research in the USA. BMJ Open. 2020; 10(1):e029852. 10.1136/bmjopen-2019-029852 PMC7044992 31959600

[13] Garofalo R , Deleon J , Osmer E , Doll M , Harper GW . Overlooked, misunderstood and at‐risk: exploring the lives and HIV risk of ethnic minority male‐to‐female transgender youth. J Adolesc Health. 2006; 38(3):230–6. 16488820 10.1016/j.jadohealth.2005.03.023

[14] Bockting W , MacCrate C , Israel H , Mantell JE , Remien RH . Engagement and retention in HIV care for transgender women: perspectives of medical and social service providers in New York City. AIDS Patient Care STDs. 2020; 34(1):16–26. 31846348 10.1089/apc.2019.0067 PMC6983737

[15] AIDS Vaccine Advocacy Coalition , Treatment Action Group (TAG) . AVAC and tag statement on ethical conduct of SARS‐CoV‐2 vaccine challenge studies. AVAC; 2020.

[16] Poteat T , Wirtz AL , Reisner SL . Strategies for engaging transgender populations in HIV prevention and care. Curr Opin HIV AIDS. 2019; 14(5):393–400. 31219887 10.1097/COH.0000000000000563

[17] Rendina HJ , Talan AJ , Cienfuegos‐Szalay J , Carter JA , Shalhav O . Treatment is more than prevention: perceived personal and social benefits of undetectable = untransmittable messaging among sexual minority men living with HIV. AIDS Patient Care STDs. 2020; 34(10):444–51. 33064015 10.1089/apc.2020.0137 PMC7585600

[18] Rebchook G , Keatley JA , Contreras R , Perloff J , Molano LF , Reback CJ , et al. The transgender women of color initiative: implementing and evaluating innovative interventions to enhance engagement and retention in HIV care. Am J Public Health. 2017; 107(2):224–9. 28075641 10.2105/AJPH.2016.303582 PMC5227951

[19] Hirshfield S , Contreras J , Luebe RQ , Swartz JA , Scheinmann R , Reback CJ , et al. Engagement in HIV care among New York City transgender women of color: findings from the peer‐led, TWEET intervention, a SPNS trans women of color initiative. AIDS Behav. 2019; 25(1):20–30. 10.1007/s10461-019-02667-6 PMC7679049 31520240

[20] Marc LG , Goldhammer H , Mayer KH , Cahill S , Massaquoi M , Nortrup E , et al. Rapid implementation of evidence‐informed interventions to improve HIV health outcomes among priority populations: the E2i Initiative. Public Health Rep. 2021. 10.1177/00333549211027849 PMC9257503 34185594

[21] Michigan Department of Community Health . Annual HIV surveillance report for Michigan. 2016.

[22] Rosenberg ES , Millett GA , Sullivan PS , Del Rio C , Curran JW . Understanding the HIV disparities between black and white men who have sex with men in the USA using the HIV care continuum: a modelling study. Lancet. 2014; 1:e112–8. 10.1016/S2352-3018(14)00011-3 PMC4269168 25530987

[23] Cowan FM , Cowan FM , Davey C , Fearon E , Mushati P , Dirawo J , et al. Targeted combination prevention to support female sex workers in Zimbabwe accessing and adhering to antiretrovirals for treatment and prevention of HIV (SAPPH‐IRe): a cluster‐randomised trial. Lancet HIV. 2018;5(8):e417–26. 30030134 10.1016/S2352-3018(18)30111-5

[24] Cabecinha MA , Saunders J . HIV prevention strategies. Medicine (Baltimore). 2022;50(4):223–228.

[25] Phanuphak N , Ramautarsing R , Chinbunchorn T , Janamnuaysook R , Pengnonyang S , Termvanich K , et al. Implementing a status‐neutral approach to HIV in the Asia‐Pacific. Curr HIV/AIDS Rep. 2020; 17(5):422–30. 32725317 10.1007/s11904-020-00516-z PMC7497381

[26] Lacombe‐Duncan A , Logie CH , Newman PA , Bauer GR , Kazemi M . A qualitative study of resilience among transgender women living with HIV in response to stigma in healthcare. AIDS Care. 2020; 32(8):1008–13. 32070113 10.1080/09540121.2020.1728212

[27] Ransome Y , Thurber KA , Swen M , Crawford ND , German D , Dean LT . Social capital and HIV/AIDS in the United States: knowledge, gaps, and future directions. Soc Sci Med. 2018; 5:73–85. 10.1016/j.ssmph.2018.05.007 PMC5991916 29892697

[28] Brewer R , Hood KB , Hotton A , Moore M , Spieldenner A , Daunis C , et al. Associations between experienced HIV stigma, resulting consequences, and the HIV care continuum: moderating effects of two resilience characteristics among persons living with HIV (PLWH) in Louisiana. J Racial Ethn Health Disparities. 2020;9:9–22. 33211250 10.1007/s40615-020-00925-1 PMC7676401

[29] Sevelius JM , Neilands TB , Dilworth S , Castro D , Johnson MO . Sheroes: feasibility and acceptability of a community‐driven, group‐level HIV intervention program for transgender women. AIDS Behav. 2020;24(5):1551–9. 31562572 10.1007/s10461-019-02683-6 PMC9148587

[30] Operario D , Gamarel KE , Iwamoto M , Suzuki S , Suico S , Darbes L , et al. Couples‐focused prevention program to reduce HIV risk among transgender women and their primary male partners: feasibility and promise of the couples HIV intervention program. AIDS Behav. 2017; 21(8):2452–63. 27334464 10.1007/s10461-016-1462-2 PMC5179320

[31] Kuhns LM , Mimiaga MJ , Reisner SL , Biello K , Garofalo R . Project LifeSkills—a randomized controlled efficacy trial of a culturally tailored, empowerment‐based, and group‐delivered HIV prevention intervention for young transgender women: study protocol. BMC Public Health. 2017; 17(1):1–7. 28915919 10.1186/s12889-017-4734-5 PMC5603056

[32] Pantelic M , Sprague L , Stangl AL . It's not “all in your head”: critical knowledge gaps on internalized HIV stigma and a call for integrating social and structural conceptualizations. BMC Infect Dis. 2019;19(1):1–8. 30832613 10.1186/s12879-019-3704-1 PMC6399894

